# Adult “termination-of-resuscitation” (TOR)-criteria may not be suitable for children - a retrospective analysis

**DOI:** 10.1186/s13049-016-0328-y

**Published:** 2016-12-07

**Authors:** Victoria Maria Rotering, Sonja Trepels-Kottek, Konrad Heimann, Jörg-Christian Brokmann, Thorsten Orlikowsky, Mark Schoberer

**Affiliations:** 1 Klinik für Kinder- und Jugendmedizin, Sektion Neonatologie, Uniklinik RWTH Aachen, Pauwelsstraße 30, 52074 Aachen, Germany; 2 Zentrale Notaufnahme, Uniklinik RWTH Aachen, Pauwelsstraße 30, Aachen, 52074 Germany

**Keywords:** Out-of-hospital-cardiac-arrest, Out-of-hospital-resuscitation, Termination-of-resuscitation, Cardiopulmonary resuscitation, Ethics, End of life, Paediatric intensive care medicine

## Abstract

**Background:**

Only a small number of patients survive out-of-hospital-cardiac-arrest (OHCA). The duration of CPR varies considerably and transportation of patients under CPR is often unsuccessful. Termination-of-resuscitation (TOR)-criteria aim to preclude futile resuscitation efforts.

Our goal was to find out to which extent existing TOR-criteria can be transferred to paediatric OHCA-patients with special regard to their prognostic value.

**Methods:**

We performed a retrospective analysis of an eleven-year single centre patient cohort. 43 paediatric patients admitted to our institution after emergency-medical-system (EMS)-confirmed OHCA from 2003 to 2013 were included. Morrison’s BLS- and ALS-TOR-rules as well as the Trauma-TOR-criteria by the American Association of EMS Physicians were evaluated for application in children, by calculating sensitivity, specificity, negative and positive predictive value for death-, as well as survival-prediction in our cohort.

**Results:**

26 patients achieved ROSC and 14 were discharged alive (*n* = 7 PCPC 1/2, *n* = 7 PCPC 5). Sensitivity for BLS-TOR-criteria predicting death was 48.3%, specificity 92.9%, the PPV 93.3% and the NPV 46.4%. ALS-TOR-criteria for death had a sensitivity of 10.3%, specificity of 100%, a PPV of 100% and an NPV of 35%.

**Conclusion:**

Retrospective application of the BLS-TOR-rule in our patient cohort identified the resuscitation of one later survivor as futile. ALS-TOR-criteria did not give false predictions of death. The proportion of CPRs that could have been abandoned is 48.2% for the BLS-TOR and only 10.3% for the ALS-TOR-rule. Both rules therefore appear not to be transferable to a paediatric population.

## Background

The incidence for paediatric out-of-hospital-cardiac-arrests (OHCA) is rather uniform throughout developed countries with 7.3-9.1-cases per 100 000 person-years [1–5].

Survival rates for paediatric and adult OHCAs are equally poor. Two to ten percent of the children who are resuscitated in the prehospital setting survive. 50% of these without neurological deficits [6, 7].

Prospects of survival are determined by the duration of untreated cardiopulmonary arrest, witnessing, potentially reversible causes, duration of cardiopulmonary resuscitation (CPR), return-of-spontaneous circulation (ROSC), heart rhythm, blood-pH, therapy and the perceptibility of definite life-extinction [8–11]. Termination-of-resuscitation (TOR)-criteria use one or more of these determinants to predict futility [12–15].

TOR-criteria have been established in the treatment of traumatic-cardiopulmonary-arrests (TCPA) in about half of US-states. The 2015 European Resuscitation Council (ERC)-Guidelines strongly advocate for their use [8, 12]. They intend to ensure a consistent, less subjective decision-making in resuscitations [12].

Validated criteria exist only for adult OHCAs because children are routinely excluded from TOR-protocols [13, 16–18]. However, early identification of futile cases is as desirable for paediatric patients. It appears that children are resuscitated more “aggressively” than adults although their prospect of survival is not better [6, 9, 19].

One reason for the reluctance to use TOR-rules in children, is a lack of empirical data regarding the safe transferability: only one study has examined the eligibility of TOR-criteria for children [20]. Therefore, we retrospectively analysed all paediatric OHCA-patients admitted to RWTH Aachen University Hospital in eleven years.

Our goal was to find out, to which extent TOR-criteria can be transferred to paediatric OHCA-patients with special regard to prognostic validity.

## Methods

### Study-design and -population

The study was approved by the local ethics committee of our institution (case number 173/14). The Institutional Review Board waived the need for informed consent as the study is of a retrospective design.

We performed a retrospective analysis of an eleven-year single centre patient cohort.

All consecutive patients younger than 18 years admitted to our institution after out-of-hospital- resuscitation (OHR) between 1st January 2003 and 1st January 2014 were tested for eligibility.

Included were patients with cardiac arrest confirmed by emergency-medical-system (EMS)-personnel. Excluded were children with respiratory arrest only or ROSC prior to EMS-arrival. Patients who were pronounced dead in the pre-hospital setting could not be included into this study since they are not admitted to our institution and data were not accessible.

We examined established adult TOR-rules under the hypothesis that they would have been applied during the CPR of the patients who are included. Primary endpoint was the (false) prediction of death in later survivors. Secondary endpoints were the neurocognitive outcome in these survivors measured as Paediatric Cerebral Performance Catetgory (PCPC) and the proportion of futile resuscitations in which TOR-criteria would have predicted death. Characteristics of OHCA and resuscitations were recorded and are presented in accordance with Utstein reporting rules [21].

The Paediatric Cerebral Performance Category (PCPC) by Fiser graduates neurological function into 6 categories: PCPC 1 represents a normal neurological function, PCPC 6 a braindead patient. PCPC 2 is a mild disability with a minor neurological handicap that does not interfere with daily activity. Moderate disability is a PCPC 3 (developmental milestones below 10th percentile and attends special classes because of cognitive deficits) and PCPC 4 is a severe disability (extensive dependence on others for daily activities and unable to attend school). PCPC 5 represents a patient who is in a vegetative state (VS), defined as any degree of coma without fulfilling the criteria for brain death. Criteria include no interaction with the environment even if awake, no cerebral responsiveness or evidence of cortical function and no reaction to verbal stimuli [21].

### Setting and institution

RWTH Aachen University Hospital is an academic tertiary care centre. 225000 patients are treated every year. 250000 inhabitants live in the catchment area of 160skm. 35000 of these are under 18 years.

### Emergency system

German EMS regularly provide teams with one trained emergency physician and at least two paramedics for severe preclinical emergencies. The prevailing concept in the German EMS is “Treat and Run”, meaning the immediate stabilisation of vital parameters on scene to secure the patient’s transportability. For Aachen and the federal state of Nordrhein-Westfalen the legally enforced arrival time is eight minutes within municipalities and twelve minutes within rural areas.

Since our institution is the only tertiary care centre in the region covering the full spectrum of traumatology including head injuries and burns as well as having a catchment area that is almost equally urban and rural, study settings were representative for the population of interest.

### TOR-criteria

Criteria for an early identification of hopeless cases in preclinical resuscitation have to be kept simple and ought to be absolutely reliable. Understanding these criteria as a statistical test, the test’s perception on prediction of death or survival determines the statistical figures for reliability: If death is predicted, specificity and positive predictive value (PPV) both need to equal 100%. PPV states how many patients in whom the rule would advise TOR (corresponding to a positive test) will actually die.

If the test predicts survival, sensitivity and negative predictive value (NPV) both need to equal 100%.

#### Established TOR-rules

##### Morrison-rule

Verbeek et al. published variables that were retrospectively predictive for death [18].

For prospective validation of Verbeek’s variables, Morrison et al. published a study about TOR during basic-life-support (BLS).

According to the BLS-TOR rule, resuscitation can be terminated when all three following negative predictors are present: no defibrillation performed, no ROSC before transport and arrest not witnessed by EMS-personnel. Morrison et al. found a specificity of 90.2% (95% CI 88.4-91.8) and a PPV for death of 99.5%. Survival was reported for 0.5% of the patients, in whom the rule would have allowed TOR. Sensitivity for death was 64.4% (95% CI 61.6-67) [16].

In 2007 Morrison et al. extended the BLS-TOR-criteria to generate TOR-criteria for advanced-life-support (ALS). According to the ALS-TOR-rule, a resuscitation may be terminated when none of the five following positive criteria for survival is met:

1. witnessed by bystanders
2. witnessed by EMS-personnel
3. bystander-CPR performed
4. shock delivered
5. ROSC before transport

Sensitivity and NPV for survival were 100% (95% CI 99.9-100).

According to the ALS-TOR-rule 32% (95% CI 30–33) of the patients were to be pronounced dead on scene. The PPV for survival was 7% (95% CI 6.6-8.1).

Applying the BLS-TOR-rule on the same cohort of patients, sensitivity and NPV for survival both equal 100% (95% CI 99.9-100) and 48% of the patients could have been pronounced dead on scene.

Other studies confirmed the reliability on various cohorts [14, 17, 22–25].

BLS-criteria find their statistical correspondence for security in the PPV. ALS-criteria on the other hand, focus on absence of positive predictors, so that NPV is the decisive parameter.

##### Trauma-TOR-rule

In the US there are very few studies analysing TOR-criteria on paediatric patients because children are usually not included into TOR-protocols [8, 18].

Capizzani et al. [20], analysed TOR-criteria for resuscitations due to trauma on a paediatric cohort. The criteria originally also exclude paediatric patients and were published by the American Association of EMS Physicians and the Committee on Trauma of the American College of Surgeons [6, 26]. The criteria are: CPR-duration greater than 15 min, no pupillary reactions, absent pulse and an “unorganized ECG” (asystole, bradycardic idioventricular rhythm, severe bradycardia and non-sinus-pulseless electrical activity).

### Data-processing and statistical analysis

Data were collected from EMS and PICU charts (electronic and paper-bound) and part of the data for patients of the years 2011 to 2013 from the German Resuscitation Registry. Missing data were excluded from statistical analysis.

Chart abstraction was performed systematically and Utstein-data were directly transmitted from paper charts to spreadsheets [21]. Data completeness and correctness was thoroughly rechecked by a second investigator.

The data collector was trained in Utstein-style reporting so consistency in definitions is guaranteed.

The criterion of “witnessing” was defined uniformly when the child was not alone during arrest and “bystander-CPR” as any effort to restore circulation.

We calculated sensitivity, specificity, NPV and PPV for death-, as well as survival-prediction on our cohort. Data collection was in accordance with Utstein-guidelines [27]. Calculation of confidence intervals and odds ratio was performed with statistical software SAS Enterprise Guide, SAS Institute Inc., Cary, NC, USA, 9.4.

## Results

### Characteristics of study subjects

**Fig. 1:**
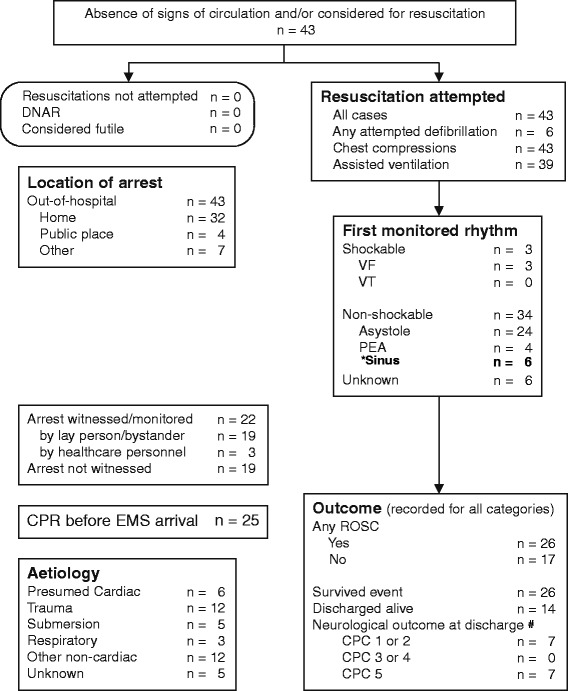
Utstein template for paediatric patient cohort in Aachen. * not part of the original version, # measured as PCPC in our cohort

Of 75 paediatric patients with OHR and consecutive hospital admission, 43 cases with cardiac arrest confirmed by emergency-medical-system (EMS)-personnel were included. 32 patients were excluded, 19 because of respiratory arrest without cardiac arrest, 13 because of ROSC prior to EMS-arrival. Of the 43 patients with EMS-confirmed cardiac arrest 17 patients were admitted under ongoing CPR, 26 after ROSC (Fig. 1). 26 patients were admitted to our paediatric intensive care unit (PICU) while 17 patients died in the Emergency Department. Of the 26 patients, who were transferred to the PICU, 14 patients were discharged alive (PCPC 1: *n* = 6 (14%), PCPC 2: *n* = 1 (2.3%), PCPC 5: *n* = 7 (16.3%)) and 12 died on the PICU (27.9%). None of the patients had an advance directive or do not attempt resuscitation (DNAR) code prior to the event. The average age of our patients was 6.03 years. 21 patients (48.8%) were male. 15 had relevant comorbidity (34.9%). Affected were seven survivors, among these one child with a complex congenital heart defect (due to a fetal alcohol syndrome), one child with multiple chromosomal defects, one former gemini-preterm infant and one former preterm infant with a congenital heart defect (Cantrell-Pentalogy), one patient with hypertophic cardiomyopathy, one child with epilepsy and one with mental retardation of unknown origin.

Eight of the non-survivors had comorbidities (one child with a Pierre-Robin sequence, two former preterm infants, one child with transposition of the great arteries, one former preterm infant with Long QT Syndrome, one child with alveolar rhabdomyosarcoma, one patient with multiple chromosomal microdeletions and epilepsy).

Main causes for CPR were trauma and other non-cardiac causes, such as drowning, sudden infant death syndrome (SIDS)/ acute life threatening event (ALTE) and epilepsy.

### TOR-criteria

#### Applying Morrison’s TOR-criteria

Fifteen patients met all three BLS-TOR-criteria. One among these, however, survived.

Therefore, sensitivity for the prognosis of death was 48.3% (14/29 deceased patients; 95% CI 30.1-66.5), specificity 92.9% (13/14 surviving patients; 95% CI 66.1-99.8), PPV 93.3% (14/15 patients; 95% CI 68.1-99.8 and NPV 46.4% (13/28 patients; 95% CI 27.2-66.1).

**Fig. 2:**
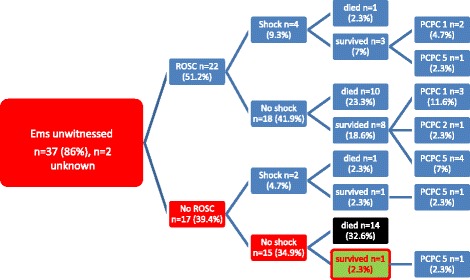
Basic-Life-Support-flow-chart for death prediction

The odds ratio was 12.1 (95% CI 1.4-105.3) (Fig. 2 and Table 1).

**Table 1:** Basic-Life-Support Termination-of-resuscitation-data for prediction of death

BLS-TOR prediction of death			
	n	%	95% CI
Sensitivity	14/29 deceased patients	48.3%	30.1-66.5
Specificity	13/14 surviving patients	92.9%	66.1-99.8
PPV	14/15 patients	93.3%	68.1-99.8
NPV	13/28 patients	46.4%	27.2-66.1
Odds ratio^a^12.13 (95% CI 1.4-105.3)			

^a^ gives the OR for death if all BLS criteria are fulfilled

Three patients (7%) met all five ALS-TOR-criteria so that termination-of-resuscitation could have been considered. The sensitivity was 10.3% (3/29 deceased patients; 95% CI 2.2-27.4), specificity 100% (14/14 surviving patients; 95% CI 99.9-100), PPV 100% (3/3 patients; 95% CI 99.9-100) and NPV 35% (14/40 patients; 95% CI 20.6-51.7) in our cohort.

None of the 14 surviving children met all the criteria for survival.

**Fig. 3:**
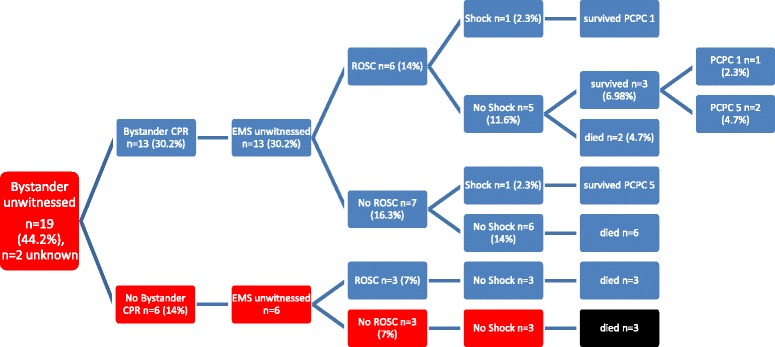
Advanced-Life-Support-flow-chart for death prediction

However, 40 patients met at least one of the criteria associated with survival (Fig. 3 and Table 2).

**Table 2:** Advanced-Life-Support Termination-of-resuscitation-data for prediction of death and survival

	ALS-TOR prediction of death			ALS-TOR prediction of survival		
	n	%	95% CI	n	%	95% CI
Sensitivity	3/29 deceased patients	10.3%	2.2-27.4	40/40 patients	100%	99.9-100
Specificity	14/14 surviving patients	100%	99.9-100	3/29 surviving patients	10.3%	2.2-27.4
Positive predictive value	3/3 patients	100%	99.9-100	14/40 patients	35.0%	20.6-51.7
Negative predictive value	14/40 patients	35%	20.6-51.7	3/3 patients	100%	99.9-100
Odds ratio^a^	Cannot be calculated					

^a^ OR cannot be given because denominator equals zero

##### Applying trauma-TOR-criteria by the American Association of EMS physicians and the committee on trauma of the American College of Surgeons

We compared Capizzani’s rules to our own cohort even though they are only intended for trauma patients, while only twelve of our patients had a traumatic cardiac arrest. We tested how many patients received CPR for more than 15 min and had fixed pupils upon EMS arrival. Mean CPR-duration was 19.3 ± 19.5 min in survivors (duration known for *n* = 10) and 37.9 ± 28.8 min in patients who died (duration known for *n* = 16).

Of those patients receiving CPR for more than 15 min and having fixed pupils, 17 died and one had PCPC 5.

CPR was performed for more than 15 min while pupillary response remained positive in three patients. One patient survived without neurological defect, the other two survived with PCPC 5.

**Fig. 4:**
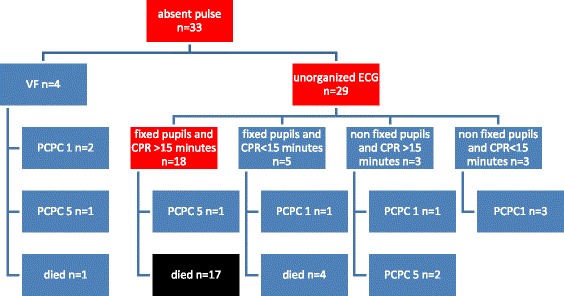
Trauma-Termination-of-resuscitation criteria (*n*=10 Patients excluded from analysis for unknown CPR-duration)

Four children died after receiving CPR for less than 15 min and having fixed pupils (Fig. 4 and Table 3).

**Table 3:** Trauma-Termination-of-resuscitation-criteria: prediction of death

	Patients		
	n	%	95% CI
Sensitivity	17/27 deceased patients	63%	44.8-81.2
Specificity	10/11 surviving patients	90.9%	73.9-107.9
PPV	17/18 patients	94.4%	83.9- 105
NPV	10/21 patients	47.6%	26.3-67

Thus, sensitivity was 63% (CI 95% 44.8-81.2), specificity 90.9% (CI 95% 73.9-107.9), PPV 94.4% (CI 95% 83.9- 105) and NPV 66.7% (CI 95% 26.3-69).

## Discussion

While validated TOR-criteria exist for adult OHCAs, they have not yet been established for the paediatric population [13, 16–18]. However, consistent decision-making and early identification of futile cases is no less desirable for this patient group. This is even more urgent considering that children are resuscitated more vigorously than adults although their prospect of survival is not better [6, 9, 19].

### Safety of TOR-criteria application

#### Morrison’s TOR-criteria

The Morrison-BLS-TOR-rule is explicitly mentioned in the 2015 ERC-Guideline on adult ALS and is the most commonly used and best validated TOR-rule [14, 17, 22–25]. Applied to our cohort, the rule does not result in 100% specificity and PPV. One survivor with a PCPC 5 would have been classified as “futile” by the rule.

Morrison’s ALS-TOR-criteria were 100% specific in our cohort and had a 100% PPV. Thus, no resuscitation would have been classified as futile in any of the survivors. With a sensitivity of 10.34% the rule would have only been applicable in one tenth of the patients who later died.

### Cardiac rhythm

One reason for ALS-TOR-rule’s limited sensitivity in the paediatric population is the different aetiology of cardiac arrest and presenting rhythm in children. Morrison et al. included only OHR due to a cardiac cause. Leading aetiologies in paediatric OHCA are trauma and SIDS [28]. Defibrillation has less influence on survival because ventricular fibrillation (VF) or pulseless ventricular tachycardia (VT) are rarely occurring rhythms in children [7, 20, 28].

### Transportation without ROSC

For a legally qualified pronouncement of death “in the field” without obvious signs of definite death, the presence of a physician is obligatory. In the Anglo-American system, patients without recognition of life extinct (ROLE) are routinely transported to a hospital, where the decision to continue or withdraw CPR is made by a physician. This proceeding leads to a high proportion of futile transportations and has triggered the demand for an “exception-rule” to systematically and safely identify OHCA-patients without any prospect of survival.

In Germany adults are less frequently transported with ongoing CPR, due to the common presence of a physician on scene. However, our impression is that emergency physicians tend to transport a higher proportion of children than adults under ongoing CPR, although published data are lacking.

It is therefore remarkable that we found two surviving children who were transported with ongoing CPR (*n* = 1: PCPC 1, *n* = 1: PCPC 5).

#### Trauma-TOR-criteria

Trauma-TOR-criteria failed to reach 100% in specificity (90.9%) and PPV (94.4%) for survival. However, the one survivor had PCPC 5.

### CPR-duration

In the “American College of Surgeons Committee on Trauma” joint-position-statement CPR-duration was found to be the most important predictor for outcome, showing enormous variability at the same time. The statement recommends to initiate a decision-making between family and professionals about terminating the resuscitation if there is no ROSC after 30 min and the next hospital is not reachable within 30 min [8]. Uniform recommendations regarding the maximum duration of a resuscitation do not exist. Epidemiological investigations often report resuscitation efforts of up to two hours [3, 29]. In the absence of a reversible cause and after asystole for more than 20 min despite ongoing ALS, the 2015 ERC-Guidelines recommend to consider withholding CPR [12, 30]. In our study, cumulative CPR-duration (sum of bystander and EMS-CPR) was known for 29 patients. We found two survivors among those patients with receiving CPR for more than 15 min, one with a good neurological outcome (PCPC 1 after 25 min of CPR) the other with PCPC 5. Average CPR-duration was 30.7 ± 26.9 min.

### Ethics of TOR-criteria

The central benefit of TOR-criteria is to preclude “futile” resuscitations. When the arrest happens at home or is witnessed by family members, continuing a futile resuscitation can interfere with relatives’ ability to make their farewells. This is most likely to be unfavourable for long-term coping, although no systematic research for family needs in paediatric OHCAs was performed yet [8]. Interviews among adults’ bereaved relatives uniformly report high satisfaction with the decision not to transport the patient after field-termination of unsuccessful resuscitations [31–33].

“Futility” can be doubtlessly assumed for those cases without a prospect of survival. We chose this narrowest definition of futility for our conclusions from this study. Many publications use a broader concept of futility that considers the functional quality of life in case of survival though. The 2010 ERC-Guideline states “Futility exists if resuscitation will be of no benefit in terms of prolonging life of acceptable quality”. The 2015-Guideline now adopted the more cautious WMA-definition [12]. It defines “futile medical treatment” to offer no reasonable hope of recovery, improvement or from which “the patient is permanently unable to experience any benefit” [34]. In clinical practice the interpretation of “life of acceptable quality” or “benefit” leaves families and medical professionals with irresolvable ethical conflicts. There is some agreement among professionals that a vegetative state (VS) is no satisfactory outcome for resuscitations. A survey in Norwegian EMS-personnel stated that VS was considered to be worse than death by the majority of respondents [35]. Judgement of futility in cases with severe disability with preserved awareness (PCPC 4) is probably most difficult and controversial.

Emergency personnel report a reluctance to stop paediatric resuscitations because they fear the parents’ reactions [9]. TOR-criteria might preserve particularly this group of patients from over-treatment which is motivated by emotions and a lack of knowledge rather than rational considerations [9]. Apart from that, secondary traumatisation for all persons involved can be eased.

Another argument in favour of TOR-criteria is a reduction in accident hazard because emergency transportations using warning devices are reduced.

They can also save resources that are valuable to other, less hopeless patients, ideally without restricting the prospects of the patient concerned.

## Conclusions

In summary, TOR-rules as a standardized approach are as desirable for children as for adults.

Neither Morrison’s BLS-TOR-rule, nor the TOR-rule for TPCA were absolutely predictive for death in our cohort. The one survivor whose CPR would have been classified as futile by both rules, had a poor neurological outcome (PCPC 5). The ALS-TOR-rule reached 100% for specificity and PPV, but could have been applied to only three of the 29 deceased patients.

Although the vast majority of transportations under ongoing CPR are futile [3, 29], this cannot be generally presumed: Two of our patients, transported under CPR, survived, one with a good neurological outcome (PCPC 1). We also highlight the benefits of TOR-criteria and advocate for further investigations in children as current data are scarce and a high risk in application exists.

## Limitations

The current study is limited by its small cohort and retrospective design as some data are therefore missing. The overall outcome of patients is better than reported (survival 32.5%), the likely reason being that we could only include those patients who were actually admitted to the Emergency Department and PICU of our institution, but not those who were declared dead on scene. This aspect is also important for the interpretation of the statistics. It is likely, that we overestimate the proportion of survivors. Apart from that, results may also be also influenced by the fact that all causes of cardiac arrest were included because some cardiac arrest causes are more easily reversible than others.

## Abbreviations

OHCA: Out-of-hospital-cardiac-arrest
OHR: Out-of-hospital-resuscitation
ROSC: Return-of-spontaneous circulation
TOR: Termination-of-resuscitation
EMS: Emergency-medical-system
TCPA: Traumatic-cardiopulmonary-arrests
ROSC: Return-of-spontaneous ventilation
NC ROCS: Non-confirmed return-of-spontaneous circulation
SIDS: Sudden infant death syndrome
ALTE: Acute life threatening event
ERC: European Resuscitation Council
DNAR: Do not attempt resuscitation
ROLE: Recognition of life extinct
VF: Ventricular fibrillation
VT: Ventricular tachycardia
ALS: Advanced-life-support
BLS: Basic-life-support
PPV: Positive predictive value
NPV: Negative predictive value
CPR: Cardiopulmonary resuscitation
PCPC: Pediatric cerebral performance category
VS: Vegetative state
